# Antimicrobial Activity Classification of Imidazolium Derivatives Predicted by Artificial Neural Networks

**DOI:** 10.1007/s11095-024-03699-x

**Published:** 2024-04-17

**Authors:** Andżelika Lorenc, Anna Badura, Maciej Karolak, Łukasz Pałkowski, Łukasz Kubik, Adam Buciński

**Affiliations:** 1 Department of Biopharmacy, Faculty of Pharmacy, Collegium Medicum in Bydgoszcz, Nicolaus Copernicus University in Toruń, dr A. Jurasza 2, 85-089 Bydgoszcz, Poland; 2 Department of Pharmaceutical Technology, Faculty of Pharmacy, Collegium Medicum in Bydgoszcz, Nicolaus Copernicus University in Toruń, dr A. Jurasza 2, 85-089 Bydgoszcz, Poland; 3 Department of Biopharmaceutics and Pharmacodynamics, Medical University of Gdańsk, Gen. J. Hallera 107, 80-416 Gdańsk, Poland

**Keywords:** artificial neural networks, classification, imidazolium derivatives, *klebsiella pneumoniae*, principal component analysis

## Abstract

**Purpose:**

This study assesses the Multilayer Perceptron (MLP) neural network, complemented by other Machine Learning techniques (CART, PCA), in predicting the antimicrobial activity of 140 newly designed imidazolium chlorides against Klebsiella pneumoniae before synthesis. Emphasis is on leveraging molecular properties for predictive analysis.

**Methods:**

Classification and regression decision trees (CART) identified the top 200 predictive molecular descriptors. Principal Component Analysis (PCA) reduced these descriptors to 5 components, retaining 99.57% of raw data information. Antimicrobial activity, categorized as high or low, was based on experimentally proven minimal inhibitory concentration (MIC), with a cut-point at MIC = 0.856 mol/L. A 12-fold cross-validation trained the MLP (architecture 5-12-2 with 5 Principal Components).

**Results:**

The MLP exhibited commendable performance, achieving almost 90% correct classifications across learning, validation, and test sets, outperforming models without PCA dimension reduction. Key metrics, including accuracy (0.907), sensitivity (0.905), specificity (0.909), and precision (0.891), were notably high. These results highlight the MLP model's efficacy with PCA as a high-quality classifier for determining antimicrobial activity.

**Conclusions:**

The study concludes that the MLP neural network, along with CART and PCA, is a robust tool for predicting the antimicrobial activity class of imidazolium chlorides against Klebsiella pneumoniae. CART and PCA, used in this study, allowed input variable reduction without significant information loss. High classification accuracy and associated metrics affirm the method’s potential utility in pre-synthesis assessments, offering valuable insights for antimicrobial compound design.

**Supplementary Information:**

The online version contains supplementary material available at 10.1007/s11095-024-03699-x.

## Introduction

Antibiotic resistance is a pressing concern as bacteria continue to develop resistance to antimicrobial substances employed in healthcare and industrial settings. This poses a significant threat to public health, as noted by the World Health Organization (WHO) and other reputable health organizations [1–4]. One of the most alarming microorganisms, with the ability to develop multi-resistance against antimicrobial agents, is *Klebsiella pneumoniae* [5]*.* This Gram-negative bacteria from the *Enterobacteriaceae* family was reported by the Centers for Disease Control and Prevention (CDC) as a high-risk pathogen due to its increasing rate of antibiotic resistance and potential to spread [6]. That trend of *K. pneumoniae* strains is especially dangerous in hospitals and other healthcare institutions where asepsis and antiseptics have a huge impact on patients’ outcomes and hospitalization time [7]. The problem increased due to large-scale disinfectant usage when the COVID-19 pandemic raised [8, 9]. The *K. pneumoniae's* propensity to develop resistance against popular antibiotics and disinfection agents and the ability to create biofilm forces scientists to find new ways of searching for new antimicrobial compounds [10–12].

The identification of potential drugs can be a time-consuming and resource-intensive process. One of the problems in the area of searching for new active agents is the multitude of compounds that can be synthesized, not all of which will exhibit desired properties. However, this process can be streamlined and made more efficient, by leveraging computational chemistry and machine learning (ML) techniques in the initial research stage. This approach reduces the need for significant resources such as time, money, and chemicals. The ultimate goal is to identify the most promising compounds, which can then be further developed [13–16].

Traditional QSAR models may struggle to capture complex and non-linear relationships between chemical structures and antimicrobial activity while the non-linear nature of ML algorithms can represent these intricate connections. Those methods are able to extract patterns and insights directly from data without relying on predefined equations, making them more adaptive to the nuances of bis-imidazolium compounds’ structure–activity relationships. It can also learn relevant features from the data, potentially uncovering subtle structural elements that contribute to antimicrobial activity. This is in contrast to QSAR, where feature selection is often a manual and hypothesis-driven process [17, 18].

Among various ML techniques used in drug research and development, the Artificial Neural Networks (ANNs) are one of the most promising. At the initial stages of molecular development, both 2D and 3D structures of compounds can be modeled, and subsequent to these models, molecular descriptors can be calculated, The obtained data can be used as predictors of antimicrobial activity by utilizing computational intelligence methods like ANNs [19–21]. This approach allows the preselection of potential antimicrobial compounds and the synthesis of only the most promising ones [22].

ANNs, widely employed in bioinformatics operate in a manner inspired by biological neural systems. These networks consist of interconnected artificial neurons organized into layers, and they become active under specific conditions. One prominent type of supervised ANN utilized in this field is the Multilayer Perceptron (MLP). The MLP is structured as a feed-forward network comprising a minimum of three layers of artificial neurons – input, (at least one) hidden, and output. Information flows from one layer to the next when it surpasses a predefined activation threshold determined by the activation function within each layer's neurons [23, 24].

Since MLPs are supervised neural networks with known input–output pairs, the primary objective is to minimize the discrepancy between predicted and actual outcomes which is indicator of the predictive quality of the network. Achieving this with a single learning attempt is nearly impossible. Therefore, the training process of an MLP is based on epochs, which represent repeated cycles of presenting the entire dataset to the network in order to optimize its performance. During each epoch, the strengths of connections (weights) between neurons are recalibrated (starting from initial random values) based on the errors observed in previous epochs – the network learns on its own mistakes.. This iterative process continues until the network's error reaches its minimum, enhancing its predictive accuracy [25].

The problem which can occur in this process is overfitting, which means that the network overly adjusts to the data which deprives its ability to generalize the knowledge. In this case, the network will perform well on known data but the ability to correctly predict outcomes for the new data (generalization) is limited.

The reasons that the model overfits the data include sample size, input data dimensionality, or regularization techniques. A small number of cases taking part in the learning process can lead the model to treat the irrelevant information (noise) as important characteristics of presented data. As the prospects of to increase the number of cases are mostly limited, some kind of multiple sampling, for example, different types of cross-validation (CV), is suggested to avoid this problem and to optimize the model and enhance its performance by better hyperparameters tuning [26].

Another issue is the high dimensionality of the input data, known as the ‘Curse of Dimensionality’ [27]. This term, introduced by Bellman indicates that the rising number of features in the dataset should be followed by an exponentially rising number of samples to maintain the balance in the model. Similarly to the previous issue, as the number of samples cannot be enhanced, the way to avoid overfitting caused by excessive dimensionality is dimensional reduction. One of the methods applied as solution is Principal Component Analysis (PCA) which, based on correlations between features, calculates the Principal Components (PCs) describing as much information included in original features as possible [28].

An alternative method to mitigate overfitting involves the utilization of regularization techniques, with one such approach being early stopping. This strategy involves the introduction of a separate dataset, referred to as a validation set. The validation set containing new, never presented to the network cases predicts the solution based on the network architecture previously built using the learning set. The function of the validation set is to check the network's generalization ability by comparing the errors made by ANN in each epoch. When the errors made in both sets are comparable, the learning process is continued, but if the error made in the learning set decreases and the validation set error increases, the network overfits the learning data and the process should be stopped [29].

The only set which doesn’t take part in the learning process is the test set. As the cases presented for this set weren’t revealed for it in the learning and test stages of network modeling, the test set role is to verify the ability of the developed model to generalize its knowledge for newly presented data [30].

In this paper, the authors would like to present a pre-synthesis approach for the classification of bis-imidazolium derivatives using Artificial Neural Networks in combination with other machine-learning techniques.. The mechanism of action of studied compounds is closely related to their chemical structure and physical properties. Compounds interact electrostatically with the negatively charged cell surfaces of microbes and surface active compounds easily penetrate through the protein–lipid biological membranes, causing disturbances in their structural and functional coherence. That make them potentialy active against considered in this research *Klebsiella pneumoniae* strains*.*

## Materials and Methods

### Structures, Synthesis, and Molecular Descriptors

The authors decided to investigate the 140 novel bis-imidazolium compounds (quaternary ammonium salts) as potential agents [31, 32].

**Fig. 1:**
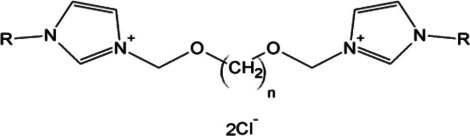
General structure of analyzed imidazolium homologs.

The structures of 140 analyzed imidazolium homologs differed in the length of the linker chain (*n* value—from 2 to 12 CH_2_ groups) and the substituent chain (Fig. 1, Table I) (full list of homologs available in Table S1 in supplementary material). Designed compounds were modeled into 2D and 3D structures as neutral compounds and the quantum mechanical Density Functional Theory (DFT), B3LYP method using Pople’s 6-31G as basis set was applied to optimization. Optimization was performed in the implicit solvent model SCRF (Self-Consistent Reaction Field) with dielectric constant set to that of water, with the use of the PCM (Polarizable Continuum Model). Calculations were performed employing Gaussian 09, Revision D.02 (Gaussian, Inc., Wallingford CT, USA), on a supercomputer cluster nods with 12-core Intel® Xeon® E5 v3 2.3 GHz processors. Geometry optimization calculations were carried out at the Centre of Informatics—Tricity Academic Supercomputer & Network. In further analysis, the 5270 molecular descriptors from 29 logical blocks were determined using Dragon 7.0 software (Talete, Milan, Italy) [33].

**Table I:** Informative System for 3,3'-(α, ω-Dioxaalkan)bis(1-Alkylimidazolium) Chlorides

Code	R
1	CH_3_
2	C_2_H_5_
3	C_3_H_7_
4	C_4_H_9_
5	C_5_H_11_
6	C_6_H_13_
7	C_7_H_15_
8	C_8_H_17_
9	C_9_H_19_
10	C_10_H_21_
11	C_11_H_23_
12	C_12_H_25_
14	C_14_H_29_
16	C_16_H_33_

The synthesis, molecules determination using nuclear magnetic resonance spectroscopy (NMR Spectra), purity examination with thin-layer chromatography (TLC), and elemental analysis of studied imidazolium compounds is described in article by Pałkowski *et al*. [34].

### Antimicrobial Activity

The minimal inhibitory concentration (MIC) value of each homolog was determined for the *K. pneumoniae* ATCC 27853 strain based on references of Clinical and Laboratory Institute (CLSI) which was also conducted and reported by mentioned above Pałkowski et.al. With reference to the standard, which was didecylmethylammonium chloride (DDAC) (a quaternary ammonium salt used for disinfection and approved by the European Economic Area as biocide [35]) with MIC = 0.856 mol/L, imidazolium compounds were categorized to high activity or low activity group. Imidazolium compounds with MICs below the MIC of the DDAC were classified in the high activity category, and those above—low activity category. This division resulted in the separation of 64 compounds classified as high activity class (45.71%) and 76 compounds classified as low activity class (54.29%) (Table S1 in supplementary material).

### Data Preprocessing

In the preprocessing stage, from the 5270 descriptors obtained in previous steps with Dragon 7.0 software the ones that were constant for all the cases and/or incomplete were deleted. That left 2711 variables for further analysis. In the next step, the data was standardized to minimize the influence of variables scaling and to make them comparable.

All the calculations in this research were conducted using STATISTICA 13, provided by StatSoft Inc., Tulsa, USA.

### Descriptors Selection

As each of the 140 examined molecules was characterized by a set of 2711 molecular descriptors, a volume of data deemed excessive for meaningful analysis within the framework of Artificial Neural Networks (ANN), so the authors opted to employ different ML techniques as a strategic approach to mitigate the challenges posed by highly probable overfitting issue.

1. Authors decided to use Classification and Regression Trees (CART) to select the best activity descriptive variables—descriptors. Based on chi-square statistics and *p*-value (*p* < 0.001) the trees have selected 200 best-fitted variables. Selected for this research 200 molecular descriptors belonged to 19 molecular blocs, and most of the descriptors were from the 2D matrix-based descriptors block (92 descriptors, 46%), 3D autocorrelations block (25 descriptors, 12.5%) and 2D autocorrelations (23 descriptors, 11.5%). (full list of selected descriptors available in Table 2 in supplementary material)
2. To limit the number of neurons and avoid data overload in the input layer, the principal component analysis (PCA) was applied. PCA is a linear method of dimension reduction, allowing to reduce the number of variables included in the network by searching the relationships between them. The correlated variables are transformed to save most of the variance of the reduced variables and create the new variable called the principal component (PC) [36] This way, 5 principal components describing 99.57% of the variability of the data contained in the 200 selected variables were extracted.

### Artificial Neural Networks calculations

Obtained in previous steps 5 PCs were used as input variables to build ANN classification models and predict, based on previously presented conditions, whether the compound presents high activity or low activity against *K. pneumoniae*. Cases were randomly divided into 3 sets: learning—98 cases (70%), validation—28 cases (20%), and test—14 cases (10%). Using the Broyden-Fletcher-Goldfarb-Shanno (BFGS) [37] learning algorithm, the software automatically modeled 500 artificial neural networks from which the one with the most optimal architecture, the best predictive ability, and the lowest error (the number of correctly classified cases) was chosen as principle ANN hyperparameters setting for further analysis.

## Results

### Model selection, metrics and evaliation

**Fig. 2:**
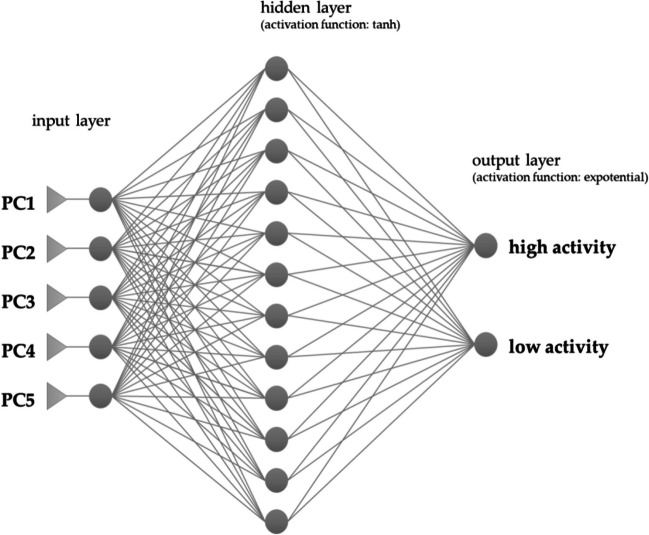
MLP 5-12-2 graph

The chosen MLP neural network was built of 5 neurons in the input layer, 12 neurons in the hidden layer, and 2 neurons in the output layer. The graph of the modeled MLP with inputs, outputs, and activation functions for hidden and input layers is shown in Fig. 2.

**Fig. 3:**
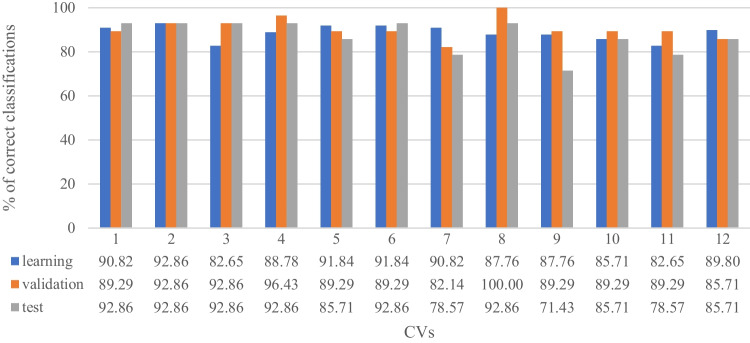
Histogram and table of classification correctness (%) of learning, validation, and test sets

As the sample size was relatively small, the 12-fold cross-validation (CV) sampling technique has been applied. The percentage of correctly classified cases in every CV fold is shown in Fig. 3.

The quality of the learning, validation, and test set was considered as an average of models obtained in the cross-validation step and established at 88.61%, 90.48%, and 86.90% respectively, which means that the model correctly classified almost 90% of cases in each set. To evaluate the quality of modeled ANN the classification metrics and statistics were calculated (Table II).

**Table II:** ANN Classification Model Metrics and Statistics

Model metrics			
Layer	Input	Hidden	Output
No. of neurons	5	12	2
Activation function		Tanh	Expotential
Model quality			
	Learning set	Validation set	Test set
Number of cases (%)	98 (70%)	28 (20%)	14 (10%)
Corectness (mean ± SD)	**88.61 ± 3.45**	**90.48 ± 4.65**	**86.90 ± 7.36**
Classification correctness			
	High activity	Low activity	All
General	64	76	140
Classified correctly	57 (89.06%)	70 (92.11%)	127 (90.71%)
Classified incorrectly	7 (10.94%)	6 (7.89%)	13 (9.29%)
Classification metrics			
	TPR	0.905	
	SPC	0.909	
	FPR	0.091	
	FDR	0.109	
	PPV	0.891	
	NPV	0.921	
	ACC	0.907	
	MCC	0.813	

To compare the performance of MLPs with and without PCA dimension reduction, the authors constructed various MLP models with different input configurations. The minimum number of inputs for MLPs without PCA was determined by the number of inputs used in MLPs with dimension reduction. The maximum number of inputs was constrained to not exceed 10% of the total cases (given the dataset size of 140 cases, the maximum number of inputs was set at 14).

The inputs were selected from the top of the list of the 200 most significant variables (according to p-value) derived initially with the CART method used for PCA dimension reduction. These variables were not subjected to further reduction and were directly used as the inputs for MLPs. The resulting models are presented in Table III.

**Table III:** Predictive Qualities Comparison of Different MLP Models

	Set quality			Error function	Activation function	
	Learning	Validation	Test		Hidden layer	Output layer
PCs MLP 5-6-2	**88.61**	**90.48**	**86.90**	**SOS**	**Logistic**	**Tanh**
MLP 5-5-2	83.25	84.23	80.95	Entropy	Expotential	Softmax
MLP 6-5-2	83.59	88.99	83.93	Entropy	Tanh	Softmax
MLP 7-16-2	82.65	89.29	83.93	Entropy	Tanh	Softmax
MLP 8-13-2	84.10	88.99	80.36	SOS	Logistic	Linear
MLP 9-13-2	82.91	89.29	82.14	SOS	Exponential	Logistic
MLP 10-16-2	84.18	90.48	79.17	Entropy	Tanh	Softmax
MLP 11-10-2	85.80	90.77	83.33	Entropy	Logistic	Softmax
MLP 12-3-2	84.27	88.69	86.31	Entropy	Tanh	Softmax
MLP 13-15-2	84.01	86.61	85.71	SOS	Tanh	Logistic
MLP 14-2-2	81.89	87.80	80.36	SOS	Logistic	Logistic

## Discussion

Current reports show that effectiveness in the search for new antimicrobial agents is estimated at around 5% [38]. Released in 2015 in ‘Review of Antimicrobial Resistance’ [39] pointed out that the number of drugs under development showing notable antimicrobial activity, especially against Gram-negative bacteria with broaden resistance is desperately low and it is estimated that among the vast of drugs under development only a few show potential against most resistant pathogens. In the mentioned review authors also pointed out that lowering the costs of antibiotics development is one of the major issues to focus on.

The majority of research is limited by financials, hence the most important problem seems to be the disproportion between the costs of the development of new drugs and the resulting benefits [40]. Implementing i*n silico* methods in the preliminary selection of modeled drugs allows excluding the compounds with low antibacterial properties, which improves the whole process by minimizing the number of synthesized compounds, reducing costs, and lowering chemical waste as the methods of searching for new antimicrobial agents are hindered by vast of financial and ecological restrictions nowadays. The combination of molecular modeling and ML methods seems to have a high potential for usage in the preclinical stage of drug research [41] limiting the needed resources. That also has been investigated on a large-scale dataset by Rahman *et al*. where they showed that the use of ML methods in the initial part of drug research can significantly increase the hit rate in searching for antimicrobial agents [42]. Research presented by Badura *et al*. shows that ANNs created to categorize 140 imidazolium compounds by their antimicrobial abilities have achieved over 90% accuracy for two bacterial strains – *E. coli* [43] and *S. aureus* [44] as well as for *C. albicans* fungus strain [45] using 20 molecular descriptors for each, but in contrast to the work presented in this paper, they worked on raw data.

The predictive ability of the selected in this research ANN allows classifying the compound to the group of high activity or low activity with almost 90% certainty. Used in the research MLP 5–12-2 neural network based on five Principal Components obtained good classification metrics with 0.905 sensitivity, 0.909 specificity, 0.891 precision, and 0.907 accuracy. The MCC which is considered as one of the most balanced classification metrics was established at 0.813. Also, FPR at the level of 0.091 shows a low probability of classifying compounds with low antimicrobial activity into the group of highly active homologs.

It should be kept in mind that appropriate feature selection is of great importance for developing accurate predictive models. The number of inputs should be adjusted to the analyzed dataset – with an increasing number of input features the risk of overfitting rises, but ANN with too small number of inputs could not gain the ability to generalize knowledge. Taking that into account, the authors used the PCA method for dimension reduction which allowed lowering the number of input variables to 5, maintaining over 99% of information carried by the 200 molecular descriptors selected with CART. This way of data condensation is widely used in computational intelligence problem-solving to avoid overfitting and increase algorithm performance speed. The concept of using PCA dimension reduction in combination with ML models is well known in medical sciences [46–49]. The paper by Chippalakatti *et al*., comparing different classification models with and without PCA dimension reduction shows that those with PC as inputs reached better performance than models based on raw data which was also shown in this paper [50]. The results depicted in Table III reveal that none of the MLP models trained on the original descriptors data outperformed the predictive quality of the MLP model trained with PCA inputs. Remarkably, the MLP model constructed with only 5 principal components encompassing the data from the 200 molecular descriptors exhibited better activity prediction than larger models based on the raw data. That result reinforces the decision to use PCA as a dimension-reduction method.

## Conclusions

The supervised MLP NN used in this research reached high values in predicting the activity class of presented imidazole compounds. The combination of different ML techniques, including dimension reduction with PCA for multivariable datasets elevated the model performance in comparison to the models based on raw data.. The application of ML methods gives scientists the opportunity to study the antimicrobial properties of compounds even in the earliest design and development stages.

Furthermore, use of several different methods such as PCA for dimension reduction to limit the number of ANN inputs, the learning process early stopping method supported by a validation set and use of additional test set to evaluate the generalization ability of the network allowed to avoid overfitting problem maintaining as best as possible performance.

We are aware that artificial neural network models are designed to predict, in this case, antimicrobial properties of certain homogenous groups of molecules, and for another group, there will be a need to build a different model. To optimize the accuracy of our models and to improve their predictive abilities, it is essential to conduct a comprehensive analysis of a wider range of compounds. The research, however, shows that the NN models demonstrate high potential as a preliminary approach to selecting designed with computational methods molecules. In our opinion, the use of ANN as a tool for the selection of compounds with antibacterial potential can have a significant impact on the performance of the initial phase of drug development.

### Supplementary Information

Below is the link to the electronic supplementary material.

ESM 1 (DOCX 42.7 KB)

## Data Availability

The datasets generated during and/or analyzed during the current study are available from the corresponding author upon reasonable request.
