# Supplementary material for: Antimicrobial Activity Classification of Imidazolium Derivatives Predicted by Artificial Neural Networks
Source: Pharm Res. 2024 Apr 17;41(5):891–8. doi: 10.1007/s11095-024-03699-x (PMC11116175; doi:10.1007/s11095-024-03699-x)
Supplement: Supplementary file 1 — (DOCX 42.7 KB) [file 11095_2024_3699_MOESM1_ESM.docx]

**Antimicrobial Activity Classification of Imidazolium Derivatives Predicted by Artificial Neural Networks**

Andżelika Lorenc ^a,*^, Anna Badura ^a^, Maciej Karolak ^b^, Łukasz Pałkowski ^b^, Łukasz Kubik ^a^, Adam Buciński ^a^

**Supplementary material**

**Table S1.** Dragon 7.0 molecular descriptors selected with CART for PCA

| No. | Block | Name | Descripton |
| --- | --- | --- | --- |
| 1 | 2D autocorrelations | ATS4v | Broto-Moreau autocorrelation of lag 4 (log function) weighted by van der Waals volume |
| 2 | 2D autocorrelations | ATS6e | Broto-Moreau autocorrelation of lag 6 (log function) weighted by Sanderson electronegativity |
| 3 | 2D autocorrelations | ATS6m | Broto-Moreau autocorrelation of lag 6 (log function) weighted by mass |
| 4 | 2D autocorrelations | ATS7m | Broto-Moreau autocorrelation of lag 7 (log function) weighted by mass |
| 5 | 2D autocorrelations | ATS7s | Broto-Moreau autocorrelation of lag 7 (log function) weighted by I-state |
| 6 | 2D autocorrelations | ATS8e | Broto-Moreau autocorrelation of lag 8 (log function) weighted by Sanderson electronegativity |
| 7 | 2D autocorrelations | ATS8v | Broto-Moreau autocorrelation of lag 8 (log function) weighted by van der Waals volume |
| 8 | 2D autocorrelations | ATSC2e | Centred Broto-Moreau autocorrelation of lag 2 weighted by Sanderson electronegativity |
| 9 | 2D autocorrelations | GATS1i | Geary autocorrelation of lag 1 weighted by ionization potential |
| 10 | 2D autocorrelations | GATS1p | Geary autocorrelation of lag 1 weighted by polarizability |
| 11 | 2D autocorrelations | JGI1 | mean topological charge index of order 1 |
| 12 | 2D autocorrelations | JGI2 | mean topological charge index of order 2 |
| 13 | 2D autocorrelations | JGI3 | mean topological charge index of order 3 |
| 14 | 2D autocorrelations | MATS1i | Moran autocorrelation of lag 1 weighted by ionization potential |
| 15 | 2D autocorrelations | MATS2i | Moran autocorrelation of lag 2 weighted by ionization potential |
| 16 | 2D autocorrelations | MATS2v | Moran autocorrelation of lag 2 weighted by van der Waals volume |
| 17 | 2D matrix-based descriptors | AVS_B(s) | average vertex sum from Burden matrix weighted by I-State |
| 18 | 2D matrix-based descriptors | AVS_D | average vertex sum from topological distance matrix |
| 19 | 2D matrix-based descriptors | AVS_Dz(e) | average vertex sum from Barysz matrix weighted by Sanderson electronegativity |
| 20 | 2D matrix-based descriptors | AVS_Dz(i) | average vertex sum from Barysz matrix weighted by ionization potential |
| 21 | 2D matrix-based descriptors | AVS_Dz(m) | average vertex sum from Barysz matrix weighted by mass |
| 22 | 2D matrix-based descriptors | AVS_Dz(p) | average vertex sum from Barysz matrix weighted by polarizability |
| 23 | 2D matrix-based descriptors | AVS_Dz(Z) | average vertex sum from Barysz matrix weighted by atomic number |
| 24 | 2D matrix-based descriptors | Chi_Dz(p) | Randic-like index from Barysz matrix weighted by polarizability |
| 25 | 2D matrix-based descriptors | Chi_Dz(v) | Randic-like index from Barysz matrix weighted by van der Waals volume |
| 26 | 2D matrix-based descriptors | H_D/Dt | Harary-like index from distance/detour matrix |
| 27 | 2D matrix-based descriptors | SM6_H2 | spectral moment of order 6 from reciprocal squared distance matrix |
| 28 | 2D matrix-based descriptors | SpAbs_Dz(e) | graph energy from Barysz matrix weighted by Sanderson electronegativity |
| 29 | 2D matrix-based descriptors | SpAbs_Dz(i) | graph energy from Barysz matrix weighted by ionization potential |
| 30 | 2D matrix-based descriptors | SpAbs_Dz(m) | graph energy from Barysz matrix weighted by mass |
| 31 | 2D matrix-based descriptors | SpAbs_Dz(p) | graph energy from Barysz matrix weighted by polarizability |
| 32 | 2D matrix-based descriptors | SpAbs_Dz(Z) | graph energy from Barysz matrix weighted by atomic number |
| 33 | 2D matrix-based descriptors | SpAD_D | spectral absolute deviation from topological distance matrix |
| 34 | 2D matrix-based descriptors | SpAD_Dz(e) | spectral absolute deviation from Barysz matrix weighted by Sanderson electronegativity |
| 35 | 2D matrix-based descriptors | SpAD_Dz(i) | spectral absolute deviation from Barysz matrix weighted by ionization potential |
| 36 | 2D matrix-based descriptors | SpAD_Dz(m) | spectral absolute deviation from Barysz matrix weighted by mass |
| 37 | 2D matrix-based descriptors | SpAD_Dz(p) | spectral absolute deviation from Barysz matrix weighted by polarizability |
| 38 | 2D matrix-based descriptors | SpAD_Dz(Z) | spectral absolute deviation from Barysz matrix weighted by atomic number |
| 39 | 2D matrix-based descriptors | SpDiam_D | spectral diameter from topological distance matrix |
| 40 | 2D matrix-based descriptors | SpDiam_Dz(e) | spectral diameter from Barysz matrix weighted by Sanderson electronegativity |
| 41 | 2D matrix-based descriptors | SpDiam_Dz(i) | spectral diameter from Barysz matrix weighted by ionization potential |
| 42 | 2D matrix-based descriptors | SpDiam_Dz(m) | spectral diameter from Barysz matrix weighted by mass |
| 43 | 2D matrix-based descriptors | SpDiam_Dz(p) | spectral diameter from Barysz matrix weighted by polarizability |
| 44 | 2D matrix-based descriptors | SpDiam_Dz(Z) | spectral diameter from Barysz matrix weighted by atomic number |
| 45 | 2D matrix-based descriptors | SpMAD_B(i) | spectral mean absolute deviation from Burden matrix weighted by ionization potential |
| 46 | 2D matrix-based descriptors | SpMAD_B(p) | spectral mean absolute deviation from Burden matrix weighted by polarizability |
| 47 | 2D matrix-based descriptors | SpMAD_B(s) | spectral mean absolute deviation from Burden matrix weighted by I-State |
| 48 | 2D matrix-based descriptors | SpMAD_B(v) | spectral mean absolute deviation from Burden matrix weighted by van der Waals volume |
| 49 | 2D matrix-based descriptors | SpMAD_L | spectral mean absolute deviation from Laplace matrix |
| 50 | 2D matrix-based descriptors | SpMax_D | leading eigenvalue from topological distance matrix |
| 51 | 2D matrix-based descriptors | SpMax_Dz(e) | leading eigenvalue from Barysz matrix weighted by Sanderson electronegativity |
| 52 | 2D matrix-based descriptors | SpMax_Dz(i) | leading eigenvalue from Barysz matrix weighted by ionization potential |
| 53 | 2D matrix-based descriptors | SpMax_Dz(m) | leading eigenvalue from Barysz matrix weighted by mass |
| 54 | 2D matrix-based descriptors | SpMax_Dz(p) | leading eigenvalue from Barysz matrix weighted by polarizability |
| 55 | 2D matrix-based descriptors | SpMax_Dz(Z) | leading eigenvalue from Barysz matrix weighted by atomic number |
| 56 | 2D matrix-based descriptors | SpMaxA_A | normalized leading eigenvalue from adjacency matrix |
| 57 | 2D matrix-based descriptors | SpMaxA_B(e) | normalized leading eigenvalue from Burden matrix weighted by Sanderson electronegativity |
| 58 | 2D matrix-based descriptors | SpMaxA_B(i) | normalized leading eigenvalue from Burden matrix weighted by ionization potential |
| 59 | 2D matrix-based descriptors | SpMaxA_B(m) | normalized leading eigenvalue from Burden matrix weighted by mass |
| 60 | 2D matrix-based descriptors | SpMaxA_B(p) | normalized leading eigenvalue from Burden matrix weighted by polarizability |
| 61 | 2D matrix-based descriptors | SpMaxA_B(s) | normalized leading eigenvalue from Burden matrix weighted by I-State |
| 62 | 2D matrix-based descriptors | SpMaxA_B(v) | normalized leading eigenvalue from Burden matrix weighted by van der Waals volume |
| 63 | 2D matrix-based descriptors | SpMaxA_L | normalized leading eigenvalue from Laplace matrix |
| 64 | 2D matrix-based descriptors | SpMaxA_X | normalized leading eigenvalue from chi matrix |
| 65 | 2D matrix-based descriptors | SpPos_D | spectral positive sum from topological distance matrix |
| 66 | 2D matrix-based descriptors | SpPos_Dz(e) | spectral positive sum from Barysz matrix weighted by Sanderson electronegativity |
| 67 | 2D matrix-based descriptors | SpPos_Dz(i) | spectral positive sum from Barysz matrix weighted by ionization potential |
| 68 | 2D matrix-based descriptors | SpPos_Dz(m) | spectral positive sum from Barysz matrix weighted by mass |
| 69 | 2D matrix-based descriptors | SpPos_Dz(p) | spectral positive sum from Barysz matrix weighted by polarizability |
| 70 | 2D matrix-based descriptors | SpPos_Dz(Z) | spectral positive sum from Barysz matrix weighted by atomic number |
| 71 | 2D matrix-based descriptors | SpPosA_B(s) | normalized spectral positive sum from Burden matrix weighted by I-State |
| 72 | 2D matrix-based descriptors | SpPosA_L | normalized spectral positive sum from Laplace matrix |
| 73 | 2D matrix-based descriptors | TI1_L | first Mohar index from Laplace matrix |
| 74 | 2D matrix-based descriptors | VE1_B(e) | coefficient sum of the last eigenvector from Burden matrix weighted by Sanderson electronegativity |
| 75 | 2D matrix-based descriptors | VE1_B(i) | coefficient sum of the last eigenvector from Burden matrix weighted by ionization potential |
| 76 | 2D matrix-based descriptors | VE1_B(m) | coefficient sum of the last eigenvector from Burden matrix weighted by mass |
| 77 | 2D matrix-based descriptors | VE2_B(p) | average coefficient of the last eigenvector from Burden matrix weighted by polarizability |
| 78 | 2D matrix-based descriptors | VE2_B(v) | average coefficient of the last eigenvector from Burden matrix weighted by van der Waals volume |
| 79 | 2D matrix-based descriptors | VE2_Dz(e) | average coefficient of the last eigenvector from Barysz matrix weighted by Sanderson electronegativity |
| 80 | 2D matrix-based descriptors | VE2_Dz(i) | average coefficient of the last eigenvector from Barysz matrix weighted by ionization potential |
| 81 | 2D matrix-based descriptors | VE2_Dz(m) | average coefficient of the last eigenvector from Barysz matrix weighted by mass |
| 82 | 2D matrix-based descriptors | VE2_Dz(Z) | average coefficient of the last eigenvector from Barysz matrix weighted by atomic number |
| 83 | 2D matrix-based descriptors | VR2_A | normalized Randic-like eigenvector-based index from adjacency matrix |
| 84 | 2D matrix-based descriptors | VR2_B(e) | normalized Randic-like eigenvector-based index from Burden matrix weighted by Sanderson electronegativity |
| 85 | 2D matrix-based descriptors | VR2_B(i) | normalized Randic-like eigenvector-based index from Burden matrix weighted by ionization potential |
| 86 | 2D matrix-based descriptors | VR2_B(m) | normalized Randic-like eigenvector-based index from Burden matrix weighted by mass |
| 87 | 2D matrix-based descriptors | VR2_B(p) | normalized Randic-like eigenvector-based index from Burden matrix weighted by polarizability |
| 88 | 2D matrix-based descriptors | VR2_B(s) | normalized Randic-like eigenvector-based index from Burden matrix weighted by I-State |
| 89 | 2D matrix-based descriptors | VR2_B(v) | normalized Randic-like eigenvector-based index from Burden matrix weighted by van der Waals volume |
| 90 | 2D matrix-based descriptors | VR2_D/Dt | normalized Randic-like eigenvector-based index from distance/detour matrix |
| 91 | 2D matrix-based descriptors | VR2_H2 | normalized Randic-like eigenvector-based index from reciprocal squared distance matrix |
| 92 | 2D matrix-based descriptors | VR2_L | normalized Randic-like eigenvector-based index from Laplace matrix |
| 93 | 2D matrix-based descriptors | Wi_Dz(v) | Wiener-like index from Barysz matrix weighted by van der Waals volume |
| 94 | 2D matrix-based descriptors | WiA_B(e) | average Wiener-like index from Burden matrix weighted by Sanderson electronegativity |
| 95 | 2D matrix-based descriptors | WiA_B(i) | average Wiener-like index from Burden matrix weighted by ionization potential |
| 96 | 2D matrix-based descriptors | WiA_B(m) | average Wiener-like index from Burden matrix weighted by mass |
| 97 | 2D matrix-based descriptors | WiA_B(p) | average Wiener-like index from Burden matrix weighted by polarizability |
| 98 | 2D matrix-based descriptors | WiA_B(s) | average Wiener-like index from Burden matrix weighted by I-State |
| 99 | 2D matrix-based descriptors | WiA_B(v) | average Wiener-like index from Burden matrix weighted by van der Waals volume |
| 100 | 2D matrix-based descriptors | WiA_H2 | average Wiener-like index from reciprocal squared distance matrix |
| 101 | 3D autocorrelations | TDB01m | 3D Topological distance based descriptors - lag 1 weighted by mass |
| 102 | 3D autocorrelations | TDB01p | 3D Topological distance based descriptors - lag 1 weighted by polarizability |
| 103 | 3D autocorrelations | TDB01s | 3D Topological distance based descriptors - lag 1 weighted by I-state |
| 104 | 3D autocorrelations | TDB01v | 3D Topological distance based descriptors - lag 1 weighted by van der Waals volume |
| 105 | 3D autocorrelations | TDB02p | 3D Topological distance based descriptors - lag 2 weighted by polarizability |
| 106 | 3D autocorrelations | TDB02s | 3D Topological distance based descriptors - lag 2 weighted by I-state |
| 107 | 3D autocorrelations | TDB02u | 3D Topological distance based descriptors - lag 2 unweighted |
| 108 | 3D autocorrelations | TDB03e | 3D Topological distance based descriptors - lag 3 weighted by Sanderson electronegativity |
| 109 | 3D autocorrelations | TDB03i | 3D Topological distance based descriptors - lag 3 weighted by ionization potential |
| 110 | 3D autocorrelations | TDB03m | 3D Topological distance based descriptors - lag 3 weighted by mass |
| 111 | 3D autocorrelations | TDB03p | 3D Topological distance based descriptors - lag 3 weighted by polarizability |
| 112 | 3D autocorrelations | TDB03r | 3D Topological distance based descriptors - lag 3 weighted by covalent radius |
| 113 | 3D autocorrelations | TDB03u | 3D Topological distance based descriptors - lag 3 unweighted |
| 114 | 3D autocorrelations | TDB03v | 3D Topological distance based descriptors - lag 3 weighted by van der Waals volume |
| 115 | 3D autocorrelations | TDB04e | 3D Topological distance based descriptors - lag 4 weighted by Sanderson electronegativity |
| 116 | 3D autocorrelations | TDB04i | 3D Topological distance based descriptors - lag 4 weighted by ionization potential |
| 117 | 3D autocorrelations | TDB04m | 3D Topological distance based descriptors - lag 4 weighted by mass |
| 118 | 3D autocorrelations | TDB04p | 3D Topological distance based descriptors - lag 4 weighted by polarizability |
| 119 | 3D autocorrelations | TDB04r | 3D Topological distance based descriptors - lag 4 weighted by covalent radius |
| 120 | 3D autocorrelations | TDB04u | 3D Topological distance based descriptors - lag 4 unweighted |
| 121 | 3D autocorrelations | TDB04v | 3D Topological distance based descriptors - lag 4 weighted by van der Waals volume |
| 122 | 3D autocorrelations | TDB05e | 3D Topological distance based descriptors - lag 5 weighted by Sanderson electronegativity |
| 123 | 3D autocorrelations | TDB05s | 3D Topological distance based descriptors - lag 5 weighted by I-state |
| 124 | 3D autocorrelations | TDB05u | 3D Topological distance based descriptors - lag 5 unweighted |
| 125 | 3D autocorrelations | TDB06s | 3D Topological distance based descriptors - lag 6 weighted by I-state |
| 126 | 3D matrix-based descriptors | Chi_RG | Randic-like index from reciprocal squared geometrical matrix |
| 127 | 3D matrix-based descriptors | H_G | Harary-like index from geometrical matrix |
| 128 | 3D matrix-based descriptors | H_G/D | Harary-like index from distance/distance matrix |
| 129 | 3D matrix-based descriptors | SM2_G/D | spectral moment of order 2 from distance/distance matrix |
| 130 | 3D matrix-based descriptors | VR2_RG | normalized Randic-like eigenvector-based index from reciprocal squared geometrical matrix |
| 131 | 3D matrix-based descriptors | WiA_RG | average Wiener-like index from reciprocal squared geometrical matrix |
| 132 | 3D-MoRSE descriptors | Mor01u | signal 01 / unweighted |
| 133 | Burden eigenvalues | SpMax1_Bh(m) | largest eigenvalue n. 1 of Burden matrix weighted by mass |
| 134 | CATS 2D | CATS2D_02_LL | CATS2D Lipophilic-Lipophilic at lag 02 |
| 135 | CATS 2D | CATS2D_03_LL | CATS2D Lipophilic-Lipophilic at lag 03 |
| 136 | CATS 3D | CATS3D_02_LL | CATS3D Lipophilic-Lipophilic BIN 02 (2.000 – 3.000 Å) |
| 137 | Charge descriptors | LDI | local dipole index |
| 138 | Connectivity indices | X0A | average connectivity index of order 0 |
| 139 | Connectivity indices | X1A | average connectivity index of order 1 |
| 140 | Constitutional indices | AMW | average molecular weight |
| 141 | Constitutional indices | GD | graph density |
| 142 | Constitutional indices | Me | mean atomic Sanderson electronegativity (scaled on Carbon atom) |
| 143 | Constitutional indices | Mv | mean atomic van der Waals volume (scaled on Carbon atom) |
| 144 | Constitutional indices | N% | percentage of N atoms |
| 145 | Constitutional indices | O% | percentage of O atoms |
| 146 | Edge adjacency indices | SM10_AEA(ed) | spectral moment of order 10 from augmented edge adjacency mat. weighted by edge degree |
| 147 | Edge adjacency indices | SM12_EA(bo) | spectral moment of order 12 from edge adjacency mat. weighted by bond order |
| 148 | Edge adjacency indices | SpMAD_EA(bo) | spectral mean absolute deviation from edge adjacency mat. weighted by bond order |
| 149 | Edge adjacency indices | SpMAD_EA(dm) | spectral mean absolute deviation from edge adjacency mat. weighted by dipole moment |
| 150 | Edge adjacency indices | SpMAD_EA(ed) | spectral mean absolute deviation from edge adjacency mat. weighted by edge degree |
| 151 | Edge adjacency indices | SpMaxA_AEA(bo) | normalized leading eigenvalue from augmented edge adjacency mat. weighted by bond order |
| 152 | Edge adjacency indices | SpMaxA_AEA(dm) | normalized leading eigenvalue from augmented edge adjacency mat. weighted by dipole moment |
| 153 | Edge adjacency indices | SpMaxA_AEA(ed) | normalized leading eigenvalue from augmented edge adjacency mat. weighted by edge degree |
| 154 | Edge adjacency indices | SpMaxA_AEA(ri) | normalized leading eigenvalue from augmented edge adjacency mat. weighted by resonance integral |
| 155 | Edge adjacency indices | SpMaxA_EA | normalized leading eigenvalue from edge adjacency mat. |
| 156 | Edge adjacency indices | SpMaxA_EA(bo) | normalized leading eigenvalue from edge adjacency mat. weighted by bond order |
| 157 | Edge adjacency indices | SpMaxA_EA(dm) | normalized leading eigenvalue from edge adjacency mat. weighted by dipole moment |
| 158 | Edge adjacency indices | SpMaxA_EA(ed) | normalized leading eigenvalue from edge adjacency mat. weighted by edge degree |
| 159 | Edge adjacency indices | SpMaxA_EA(ri) | normalized leading eigenvalue from edge adjacency mat. weighted by resonance integral |
| 160 | ETA indices | Eta_B_A | eta average branching index |
| 161 | ETA indices | Eta_beta_A | eta average VEM count |
| 162 | ETA indices | Eta_betaP_A | eta pi and lone pair average VEM count |
| 163 | ETA indices | Eta_betaS_A | eta sigma average VEM coun |
| 164 | ETA indices | Eta_epsi_A | eta average electronegativity measure |
| 165 | ETA indices | Eta_FL_A | eta average local functionality index |
| 166 | ETA indices | Eta_sh_p | eta p shape index |
| 167 | ETA indices | Eta_sh_y | eta y shape index |
| 168 | GETAWAY descriptors | HATS5s | leverage-weighted autocorrelation of lag 5 / weighted by I-state |
| 169 | Information indices | AAC | mean information index on atomic composition |
| 170 | Information indices | BIC0 | Bond Information Content index (neighborhood symmetry of 0-order) |
| 171 | Information indices | Ges | Number of symmetry classes (based on electrotopological state) |
| 172 | Information indices | HDcpx | graph distance complexity index (log function) |
| 173 | Information indices | IC0 | Information Content index (neighborhood symmetry of 0-order) |
| 174 | Information indices | IDET | total information content on the distance equality |
| 175 | Information indices | SIC0 | Structural Information Content index (neighborhood symmetry of 0-order) |
| 176 | Molecular properties | BLTA96 | Verhaar Algae base-line toxicity from MLOGP (mmol/l) |
| 177 | Molecular properties | BLTD48 | Verhaar Daphnia base-line toxicity from MLOGP (mmol/l) |
| 178 | Molecular properties | BLTF96 | Verhaar Fish base-line toxicity from MLOGP (mmol/l) |
| 179 | Molecular properties | Hy | hydrophilic factor |
| 180 | Molecular properties | MLOGP | Moriguchi octanol-water partition coeff. (logP) |
| 181 | Molecular properties | MLOGP2 | squared Moriguchi octanol-water partition coeff. (logP^2) |
| 182 | Molecular properties | PDI | packing density index |
| 183 | P_VSA-like descriptors | P_VSA_MR_1 | P_VSA-like on Molar Refractivity, bin 1 |
| 184 | RDF descriptors | RDF030i | Radial Distribution Function - 030 / weighted by ionization potential |
| 185 | RDF descriptors | RDF155s | Radial Distribution Function - 155 / weighted by I-state |
| 186 | Ring descriptors | MCD | molecular cyclized degree |
| 187 | Topological indices | CENT | centralization |
| 188 | Topological indices | Gnar | Narumi geometric topological index |
| 189 | Topological indices | Psi_e_A | electrotopological state pseudoconnectivity index - type S average |
| 190 | Topological indices | Psi_i_A | intrinsic state pseudoconnectivity index - type S average |
| 191 | Topological indices | TIE | E-state topological parameter |
| 192 | Topological indices | UNIP | unipolarity |
| 193 | Topological indices | VAR | variation |
| 194 | Topological indices | Wap | all-path Wiener index |
| 195 | Walk and path counts | MPC07 | molecular path count of order 7 |
| 196 | Walk and path counts | MPC09 | molecular path count of order 9 |
| 197 | Walk and path counts | MWC08 | molecular walk count of order 8 |
| 198 | Walk and path counts | piPC06 | molecular multiple path count of order 6 |
| 199 | Walk and path counts | piPC08 | molecular multiple path count of order 8 |
| 200 | Walk and path counts | TPC | total path count |

**Table S2.** Minimal inhibitory concentration and activity of 140 3,3'-(α, ω-dioxaalkan)bis(1-alkylimidazolium) chlorides

| object |  | | MIC | activity |  | object |  | | MIC | activity |  | object |  | | MIC | activity |  | object |  | | MIC | activity |  | object |  | | MIC | activity |
| --- | --- | --- | --- | --- | --- | --- | --- | --- | --- | --- | --- | --- | --- | --- | --- | --- | --- | --- | --- | --- | --- | --- | --- | --- | --- | --- | --- | --- |
|  |  |  |  |  |  |  |  |  |  |  |  |  |  |  |  |  |  |  |  |  |  |  |  |  |  |  |  |  |
|  | n | R |  |  |  |  | n | R |  |  |  |  | n | R |  |  |  |  | n | R |  |  |  |  | n | R |  |  |
| 1 | 2 | 1 | 33.875 | low |  | 29 | 4 | 1 | 28.468 | low |  | 57 | 6 | 1 | 3.295 | low |  | 85 | 8 | 1 | 0.919 | low |  | 113 | 10 | 1 | 27.375 | low |
| 2 | 2 | 2 | 28.468 | low |  | 30 | 4 | 2 | 26.363 | low |  | 58 | 6 | 2 | 26.360 | low |  | 86 | 8 | 2 | 1.697 | low |  | 114 | 10 | 2 | 12.711 | low |
| 3 | 2 | 3 | 13.181 | low |  | 31 | 4 | 3 | 26.360 | low |  | 59 | 6 | 3 | 22.966 | low |  | 87 | 8 | 3 | 0.788 | high |  | 115 | 10 | 3 | 11.865 | low |
| 4 | 2 | 4 | 6.590 | low |  | 32 | 4 | 4 | 22.966 | low |  | 60 | 6 | 4 | 21.576 | low |  | 88 | 8 | 4 | 2.966 | low |  | 116 | 10 | 4 | 22.249 | low |
| 5 | 2 | 5 | 5.741 | low |  | 33 | 4 | 5 | 5.394 | low |  | 61 | 6 | 5 | 10.172 | low |  | 89 | 8 | 5 | 0.178 | high |  | 117 | 10 | 5 | 0.649 | high |
| 6 | 2 | 6 | 5.394 | low |  | 34 | 4 | 6 | 1.261 | low |  | 62 | 6 | 6 | 0.597 | high |  | 90 | 8 | 6 | 0.168 | high |  | 118 | 10 | 6 | 0.316 | high |
| 7 | 2 | 7 | 2.543 | low |  | 35 | 4 | 7 | 0.308 | high |  | 63 | 6 | 7 | 0.292 | high |  | 91 | 8 | 7 | 0.079 | high |  | 119 | 10 | 7 | 0.075 | high |
| 8 | 2 | 8 | 0.597 | high |  | 36 | 4 | 8 | 0.146 | high |  | 64 | 6 | 8 | 0.278 | high |  | 92 | 8 | 8 | 0.037 | high |  | 120 | 10 | 8 | 0.036 | high |
| 9 | 2 | 9 | 0.566 | high |  | 37 | 4 | 9 | 0.069 | high |  | 65 | 6 | 9 | 0.133 | high |  | 93 | 8 | 9 | 0.071 | high |  | 121 | 10 | 9 | 0.526 | high |
| 10 | 2 | 10 | 0.538 | high |  | 38 | 4 | 10 | 0.133 | high |  | 66 | 6 | 10 | 0.253 | high |  | 94 | 8 | 10 | 0.068 | high |  | 122 | 10 | 10 | 0.259 | high |
| 11 | 2 | 11 | 0.513 | high |  | 39 | 4 | 11 | 0.063 | high |  | 67 | 6 | 11 | 0.030 | high |  | 95 | 8 | 11 | 0.259 | high |  | 123 | 10 | 11 | 0.960 | low |
| 12 | 2 | 12 | 0.981 | low |  | 40 | 4 | 12 | 0.940 | low |  | 68 | 6 | 12 | 0.233 | high |  | 96 | 8 | 12 | 0.960 | low |  | 124 | 10 | 12 | 7.420 | low |
| 13 | 2 | 13 | 1.817 | low |  | 41 | 4 | 13 | 0.433 | high |  | 69 | 6 | 13 | 0.417 | high |  | 97 | 8 | 13 | 0.883 | low |  | 125 | 10 | 13 | 6.849 | low |
| 14 | 2 | 14 | 1.680 | low |  | 42 | 4 | 14 | 0.052 | high |  | 70 | 6 | 14 | 3.125 | low |  | 98 | 8 | 14 | 0.409 | high |  | 126 | 10 | 14 | 12.721 | low |
| 15 | 3 | 1 | 29.652 | low |  | 43 | 5 | 1 | 27.375 | low |  | 71 | 7 | 1 | 16.937 | low |  | 99 | 9 | 1 | 7.117 | low |  | 127 | 12 | 1 | 13.181 | low |
| 16 | 3 | 2 | 27.375 | low |  | 44 | 5 | 2 | 25.423 | low |  | 72 | 7 | 2 | 7.117 | low |  | 100 | 9 | 2 | 6.591 | low |  | 128 | 12 | 2 | 13.180 | low |
| 17 | 3 | 3 | 25.423 | low |  | 45 | 5 | 3 | 23.730 | low |  | 73 | 7 | 3 | 6.591 | low |  | 101 | 9 | 3 | 6.590 | low |  | 129 | 12 | 3 | 2.871 | low |
| 18 | 3 | 4 | 5.933 | low |  | 46 | 5 | 4 | 2.781 | low |  | 74 | 7 | 4 | 6.590 | low |  | 102 | 9 | 4 | 2.871 | low |  | 130 | 12 | 4 | 2.697 | low |
| 19 | 3 | 5 | 11.125 | low |  | 47 | 5 | 5 | 20.942 | low |  | 75 | 7 | 5 | 1.424 | low |  | 103 | 9 | 5 | 0.345 | high |  | 131 | 12 | 5 | 0.041 | high |
| 20 | 3 | 6 | 5.235 | low |  | 48 | 5 | 6 | 4.945 | low |  | 76 | 7 | 6 | 0.669 | high |  | 104 | 9 | 6 | 0.326 | high |  | 132 | 12 | 6 | 0.077 | high |
| 21 | 3 | 7 | 0.613 | high |  | 49 | 5 | 7 | 18.74 | low |  | 77 | 7 | 7 | 0.041 | high |  | 105 | 9 | 7 | 0.154 | high |  | 133 | 12 | 7 | 0.073 | high |
| 22 | 3 | 8 | 0.581 | high |  | 50 | 5 | 8 | 0.285 | high |  | 78 | 7 | 8 | 0.038 | high |  | 106 | 9 | 8 | 0.073 | high |  | 134 | 12 | 8 | 0.139 | high |
| 23 | 3 | 9 | 0.552 | high |  | 51 | 5 | 9 | 2.120 | low |  | 79 | 7 | 9 | 0.146 | high |  | 107 | 9 | 9 | 0.278 | high |  | 135 | 12 | 9 | 4.141 | low |
| 24 | 3 | 10 | 0.271 | high |  | 52 | 5 | 10 | 0.259 | high |  | 80 | 7 | 10 | 0.017 | high |  | 108 | 9 | 10 | 0.265 | high |  | 136 | 12 | 10 | 1.978 | low |
| 25 | 3 | 11 | 0.502 | high |  | 53 | 5 | 11 | 0.062 | high |  | 81 | 7 | 11 | 0.513 | high |  | 109 | 9 | 11 | 0.981 | low |  | 137 | 12 | 11 | 3.789 | low |
| 26 | 3 | 12 | 0.480 | high |  | 54 | 5 | 12 | 0.119 | high |  | 82 | 7 | 12 | 0.491 | high |  | 110 | 9 | 12 | 3.789 | low |  | 138 | 12 | 12 | 14.537 | low |
| 27 | 3 | 13 | 7.123 | low |  | 55 | 5 | 13 | 0.425 | high |  | 83 | 7 | 13 | 0.451 | high |  | 111 | 9 | 13 | 3.492 | low |  | 139 | 12 | 13 | 6.720 | low |
| 28 | 3 | 14 | 6.596 | low |  | 56 | 5 | 14 | 1.590 | low |  | 84 | 7 | 14 | 0.833 | high |  | 112 | 9 | 14 | 6.476 | low |  | 140 | 12 | 14 | 12.498 | low |
